# Relationships between tryptophan-related gut metabolites, brain activity, and autism symptomatology

**DOI:** 10.21203/rs.3.rs-4559624/v1

**Published:** 2024-07-25

**Authors:** Lisa Aziz-Zadeh, Emeran Mayer, Jennifer Labus, Sofronia Ringold, Aditya Jayashankar, Emily Kilroy, Christiana Butera, Jonathan Jacobs, Skylar Tanartkit, Swapna Joshi, Mirella Dapretto

**Affiliations:** USC; G. Oppenheimer Center for Neurobiology of Stress and Resilience at UCLA; Institute for Genomics and Bioinformatics, University of California, Irvine; UCLA; USC; USC; USC; USC; UCLA; UCLA; UCLA; UCLA

## Abstract

Gut microbial metabolites have been theorized to play a causative role in the pathophysiology of autism spectrum disorder (ASD). This hypothesis is based on results from mechanistic preclinical studies and several correlational studies showing differences in gut microbial composition between ASD subjects and neurotypical (NT) controls. However, alterations in how the human brain interacts with the gut microbiome in ASD have not been examined. In this cross-sectional, case-control observational study, fecal metabolomics, task-based functional magnetic resonance imaging (fMRI), and behavioral assessments were obtained from 43 ASD and 41 NT children aged 8–17. The fMRI tasks were based on socio-emotional and sensory paradigms that commonly show strong evoked brain differences in ASD participants. General linear models and mediational modeling were applied to examine the links between tryptophan metabolism and evoked brain activity and behavior. Results indicated that fecal levels of specific tryptophan-related metabolites were associated with: 1) brain activity atypicalities in regions previously implicated in ASD (i.e., insula and cingulate); and 2) ASD severity and symptomatology (i.e., ADOS scores, disgust propensity, and sensory sensitivities). Importantly, activity in the mid-insula and mid-cingulate significantly mediated relationships between the microbial tryptophan metabolites, indolelactate and tryptophan betaine, and ASD severity and disgust sensitivity. To our knowledge, this is the first study to elucidate how interactions between gut metabolites and brain activity may impact autism symptomatology, particularly in functional brain pathways associated with vagal and interoceptive/emotion processing.

## 1. Introduction

**Figure 1:**
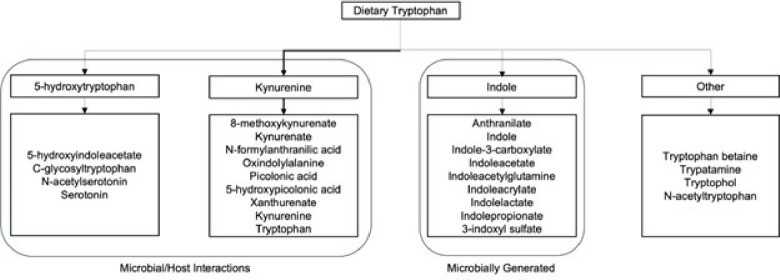
Metabolites within the tryptophan pathway *Note*. Arrow thickness represents the strength of the pathway under normal conditions.

The gut microbial ecosystem generates an estimated 40% of all metabolites in the body’s circulation, including neuroactive and inflammatory molecules (see Figure 1).^1,2^ Within the brain-gut-microbiome (BGM) system, such neuroactive gut metabolites can modulate brain activity directly or via systemic circulation or vagal and spinal afferents. Ninety percent of vagal fibers are afferents, underscoring the magnitude of information from the gut microbiome and vagal signals to the brain.^3,4^ Together, these regulatory signals from the gut microbiome can influence socio-emotional and sensory processing, cognition, and behavior.^5–8^ Pre- and postnatal microbial and neural disruptions may have profound effects on the development of the enteric and central nervous system.^9^ Such disruptions have been linked to the etiology of neurodevelopmental disorders, such as autism spectrum disorder (ASD).^10–14^

Indeed, longitudinal studies in ASD indicate that gut microbial dysbiosis can be found in infancy^15^ and persist through adulthood.^16,17^ Early life gut dysbiosis may be associated with compromised integrity of barriers within the BGM system, contributing to the development of neurodevelopmental disorders, such as ASD.^14,18,19^ Further, cross-sectional human studies indicate that gut microbiome and metabolite alterations correlate with ASD symptoms (i.e., difficulties in socio-emotional behavior and sensory perception), which can be ameliorated with probiotics or fecal microbiota transplants from neurotypical (NT) children.^20–23^ The high prevalence of gastrointestinal symptoms in ASD (46–84%) further underscores the gut as an important focus in ASD research.^3,5,24,25^

Of the gut microbial metabolites that may be most relevant to ASD pathophysiology, there has long been an interest in those in the serotonin pathway (Figure 1). Roughly 30% of autistic individuals show elevated blood serotonin levels, which have been linked to dysregulation of symptoms such as mood, appetite, and social interactions via direct vagal signaling.^26–28^ In addition, autistic individuals with hyper-serotonemia are more likely to experience GI issues.^29,30^ Surprisingly, reducing dietary tryptophan, which is the precursor for 95% of the body’s serotonin, has been associated with increased ASD symptomatology.^31–34^ Gut microbes also play an important role in the metabolism of dietary tryptophan into indoles and kynurenine, which, in contrast to serotonin, can cross the blood-brain-barrier. These metabolites can have neuroprotective or neurotoxic effects especially during critical periods of development, influencing brain microstructure, activity and development.^5,35,36^ Thus, there is evidence that dysregulations in the tryptophan metabolism pathway may influence ASD symptomatology, at least in a subset of individuals.

However, to date, there are no human ASD studies that have looked at interactions between gut metabolites and brain activity, and their impact on ASD symptomatology. In this cross-sectional study, 43 ASD and 41 neurotypical (NT) youth (aged 8–17) underwent functional MRI studies (fMRI), fecal metabolomics focused on the tryptophan pathway, and comprehensive clinical and behavioral assessments. Brain imaging tasks focused on interoceptive, emotional, and sensory processing regions of interest (ROIs) based on prior fMRI studies in children with ASD (see Methods). The fMRI tasks all involved socio-emotional processing (processing emotions, facial expressions, others’ somatosensory experiences); such socio-emotional processing has been shown to be modulated by tryptophan metabolites, like serotonin.^31,32,37^ Our primary hypothesis was that tryptophan-related gut metabolites would correlate with ASD symptomatology, and with atypical brain activity in regions consistently implicated in ASD (i.e., insular subregions, pregenual anterior and mid-cingulate). We additionally explored the hypothesis that task-based brain activity would mediate metabolite and behavioral relationships.

## 2. Results

### 2.1 Behavioral Measures.

Independent samples t-tests and Fisher’s exact tests were used to determine significant ASD-NT differences in demographic and behavioral variables. As shown in Supplemental Table 1, compared to the NT group (N=41), the ASD group (N=43) showed significantly higher: body mass index (BMI), prenatal antibiotic use, gastrointestinal (GI) symptoms, sensory sensitivities, attention deficit/hyperactivity disorder (ADHD) symptoms, disgust sensitivity, and social difficulties. Additionally, the ASD group showed significantly lower scores on the full-scale intelligence quotient (FSIQ) and sleep quality (all *p*s<.05).

### 2.2 Brain-Metabolite Correlations in the ASD Group.

**Figure 2:**
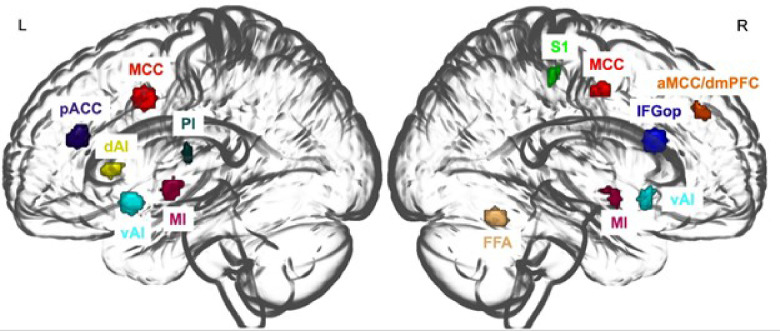
Regions of Interest (ROIs) based on between-group differences in fMRI tasks *Note*. See Supplemental Table 3 for peak MNI coordinates for each ROI. While our predominant focus was on subregions of the insula and cingulate due to their involvement in interoceptive and emotional processing, we additionally considered other ROIs with significant ASD vs. TD differences in our fMRI tasks and from prior ASD studies. Please see Methods for how ROIs were selected. R: Right; L: Left; Insular subregions (dAI: left dorsal anterior insula; vAI: ventral anterior insula; MI: mid-insula; PI: posterior insula); Cingulate subregions (pACC: pregenual anterior cingulate cortex; MCC: mid-cingulate cortex; aMCC/dmPFC:anterior mid-cingulate cortex/ dorsal medial prefrontal corte); IFGop: inferior frontal gyrus, pars opercularis; S1: primary somatosensory cortex.

We focused on brain regions involved in interoceptive, vagal, and emotion processing, as these may be especially impacted in gut-brain interactions. For brain regions of interest (ROIs), we focused on regions that showed significant differences in ASD based on prior studies and our fMRI tasks (tasks included: processing others’ facial expressions/actions; disgust processing; processing somatosensory experiences). As Figure 2 shows, ROIs included the: cingulate cortex (bilateral mid-cingulate, pregenual anterior cingulate, anterior mid-cingulate cortex/dorsal medial prefrontal cortex), bilateral insula (anterior, dorsal anterior, mid, ventral, posterior), right fusiform face area (FFA), right inferior gyrus pars opercularis (IFGop), and right primary somatosensory cortex (S1). See Methods (4.6.2) for detailed analysis of the functional imaging tasks.

General linear models (GLMs) were applied to assess associations between brain regions showing differences on the evoked tasks with metabolites, controlling for age, sex, FSIQ, and BMI. See Table 1 for metabolite-brain association results for the ASD group (and *Supplemental Table 6* for NT results). During facial expression processing, increased activity in the right IFGop was significantly associated with higher levels ofanthranilate, while decreased activity in the left mid-cingulate cortex (MCC) was significantly associated with higher levels of tryptophan betaine. Activity in the left MCC when processing hand actions was significantly associated with increased abundance of n-acetyltryptophan. Additionally, higher left MCC activity during processing of non-emotional faces was significantly associated with increased c-glycosyl tryptophan.

For the disgust processing task, significant ROI-metabolite correlations primarily involved insular subregions. Notably, lower levels of indolelactate were significantly associated with increased activity in the right mid-insula when viewing disgusting foods. When viewing disgust facial expressions, increased activity in the left dorsal anterior insula was associated with higher levels of kynurenate.

For the somatosensory task, increased levels of indole-3-carboxylate were significantly associated with increased activity in right S1 and anterior mid-cingulate cortex/ dorsomedial prefrontal cortex (aMCC/dmPFC) when processing non-social touch. By contrast, decreased levels of indole-3-carboxylate were associated with activity in the left posterior insula when processing social touch.

Brain-behavioral association results within the ASD group are shown in Table 2. Of note, increased left MCC activity related to non-emotional facial processing was positively associated with disgust sensitivity across various stimuli conditions. Further, when looking at disgusting foods, decreased activity in the right mid-insula was significantly associated with autism severity (ADOS-2 RRB and ADOS-2 total score), and activity in the left ventral anterior insula was associated with restricted, repetitive, and stereotyped patterns of behavior (ADI-R RRB).

### 2.3 Behavioral Associations with Select Tryptophan Metabolites within the ASD Group.

The above results (Section 2.2) indicated that 7 tryptophan metabolites were significantly correlated with alterations in brain activity in ASD. We applied GLMs to investigate how these 7 metabolites may also be related to behavior in the ASD group, while controlling for age, sex, BMI, and FSIQ. As Table 3 shows, we found large effect size associations between metabolites and behavioral variables. Higher levels of N-acetyltryptophan were significantly correlated with autism severity as measured by the Autism Diagnostic Interview-Revised (ADI-R). Additionally, higher levels of anthranilate were significantly correlated with greater quality of sleep.

### 2.4 Mediation Analysis.

**Figure 3:**
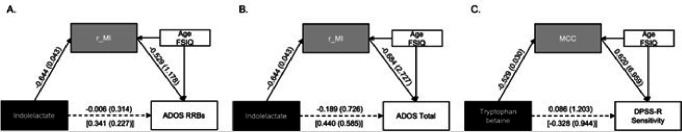
Significant mediation models in the ASD group. *Note*. Mediation models within the ASD group, with the brain as the mediator between specific metabolites and behavior. For each mediation model, the figure contains the standardized beta and standard error: Std. β (SE) for direct effects. The indirect effect between the metabolite and behavior is listed below the arrow in brackets. **A:** Right mid-insula: disgusting foods vs. rest, indolelactate, and ADOS RRB. **B:** Right mid-insula: disgusting foods vs rest, indolelactate, and ADOS total score. **C:** Left mid-cingulate: non-emotional faces vs rest, tryptophan betaine, and disgust sensitivity, ADOS: Autism Diagnosis Observation Schedule; ADI-R: Autism Diagnostic Interview-Revised; RRB: Restricted and Repetitive Behaviors; DPSS-R: Disgust Propensity and Sensitivity Scale - Revised; r_MI: right mid-insula; l_ MCC: mid-cingulate cortex.

We conducted exploratory mediation analyses, with the brain as the mediator between microbiome-behavior relationships. As Figure 3 shows, mediation models (controlling for brain-related variables: age and FSIQ) indicated significant mediating effects for the right mid-insula and the left mid-cingulate. Specifically, activity in the right mid-insula during disgust processing mediated the relationship between indolelactate and autism severity for ADOS RRB score (indirect effect: Std. β=0.341, SE=0.227, 95% CI [0.016,0.967] and ADOS total score (indirect effect: Std. β=0.440, SE=0.585, 95% CI [0.005, 0.984]). Additionally, activity in the left mid-cingulate during facial expression processing mediated the relationship between tryptophan betaine and disgust sensitivity (indirect effect: Std. β=−0.328, SE=−0.944, 95% CI [−0.610–0.075]).

## 3. Discussion

The current results confirmed our main hypotheses that tryptophan-related gut metabolites show significant associations with known ASD brain atypicalities and symptomatology, with medium to large effect sizes. Additionally, we found that brain activity mediates the relationship between metabolites and behavior. To our knowledge, this is the first study to examine relationships between gut metabolites and neural activity in ASD youth, and to report that brain activity mediates the relationship between tryptophan-metabolites and ASD symptomatology. These findings represent a novel and important step toward a better mechanistic understanding of ASD.

In this study, we focused on activity in brain regions (ROIs) previously implicated in ASD, particularly neural regions important for interoceptive, vagal, disgust, and socio-emotional processing.^38–42^ Indeed, activity in these ROIs was significantly related to autism symptomatology and severity (*see Supplemental Table 4*). Importantly, or the first time, we show that in ASD, activity in these ROIs is significantly correlated with abundance of gut-derived metabolites in the tryptophan pathway, including indoles and serotonin (see Fig. 1), which have been implicated in previous ASD research.^43,44^ Specifically, we found that tryptophan metabolites are significantly correlated with task-based brain activity (left pregenual anterior and middle cingulate cortex, insular subregions, right S1) and with autism severity and symptomatology.

Notably, in line with our second hypothesis, we found that in ASD, brain activity in the mid-cingulate associated with social processing modulated the relationship between tryptophan betaine and disgust sensitivity. Tryptophan-related metabolites are important modulators of vagal activity.^45^ Their altered abundance in the gut has been significantly linked to a number of psychological, neurological, and medical symptoms, such as memory loss, long COVID, depression, sleep disturbances, and anxiety.^45,46^ Thus, the altered abundance of tryptophan-related metabolites may modify vagal signaling or directly activate brainstem nuclei, influencing upstream MCC brain activity during socio-emotional tasks and impacting emotional processing. The current data helps provide a mechanistic explanation for common ASD differences in brain activity (MCC, insula) and behavior (disgust sensitivity).^42,47^

Further, in the ASD group, we specifically found several correlations between indole metabolites, brain activity (insular subregions, aMCC/dmPFC, IFGop, and S1), and symptomatology (autism severity [ADI-R RSI] and alexithymia). Most interestingly, supporting our second hypothesis, we found that disgust processing activity in the right mid-insula mediates the relationship between indolelactate and two measures of autism severity (ADOS total score and ADOS RRBs). This is particularly noteworthy given that: 1) disgust processing is strongly related to interoceptive processing, making it particularly suited for gut metabolite influence; 2) autistic children commonly have differences in disgust processing;^42,47^ and 3) the mid-insula is a hub of interoceptive, emotion, and chemosensory processing, and is particularly known to show atypical activity and connectivity in ASD.^47–49^ Thus, these data strongly support the notion that tryptophan-related gut metabolites impact brain function, contributing to some ASD behavioral symptomatologies.

Prior studies have also found that differences in levels of indole metabolites in ASD correlate with autism symptomatology.^23,50,51^ For example, Needham et al. (2021) found significant correlations between fecal levels of several indoles, such as indolepropionate, indole, n-formlyanthranalic acid, and indole-3-carboxylate and ASD severity (ADI-R, ADOS).^23^ The current results expand upon these previous findings by adding that activity in the right mid-insula may be the important mediator between indole-behavior relationships in ASD. Thus, these data provide a mechanistic account of prior metabolite-brain relationships.

Interestingly, we found that a history of prenatal antibiotic exposure was significantly higher in the ASD group. Prenatal maternal antibiotic exposure has been shown to affect the maternal microbiome, which can influence the fetal brain’s exposure to altered maternal microbial metabolites. This prenatal mechanism has been hypothesized to play a role in the etiology of ASD.^13,52^ However, in a post-hoc analysis using independent samples t-tests, we found no significant differences in metabolite abundances in the tryptophan pathway between ASD participants with and without prenatal antibiotic exposure; further studies are needed on this topic.

There are several limitations to the current study. We had a predominantly male sample with strict inclusion criteria for the fMRI component (right handedness, FSIQ > 79, 8–17-year-olds, exclusion of other neuropsychiatric and neurological disorders), limiting sample heterogeneity and size. To account for this, we utilized a hypothesis-driven approach considering only metabolites within the tryptophan pathway and a-priori brain ROIs to limit the number of comparisons. Additionally, multiple comparisons corrections were used in all GLM analyses. Further, the study design was cross-sectional and did not allow the assessment of a causal relationship of the gut microbiome to brain or behavior. Future research should include more heterogeneous larger samples and longitudinal designs aimed at studying critical periods of development, and analysis of broader metabolite pathways to better understand relationships between the brain, gut microbiome, and behavior in ASD.

We note that a study by Yap and colleagues (2021) found that microbiome differences between ASD and NT samples were driven by less diverse diets, likely due to significantly restricted ASD food preferences, and thus cautioned against claiming a causative role of the microbiome in ASD pathophysiology.^53^ However, in the current study, a post-hoc analysis using independent samples t-tests found no group differences in metabolite concentrations between the diet types and no associations with diet between groups or significant correlations between diet and metabolites. Thus, in this sample, it does not seem that diet was a major driver of metabolite correlations. Further, the current study addresses many critiques of prior studies suggested by Yap et al. (2021);^53^ we included microbiome-relevant factors as covariates (age, sex, BMI), used multiple comparisons corrections in all analyses, excluded participants on antibiotics, prebiotics, and probiotics, and measured and considered other factors potentially affecting the microbiome (sleep, delivery method, GI issues, medication). Nevertheless, future research should include more in-depth dietary analyses of participants (and their mothers during the prenatal stage), using tools such as the foodMAST platform’s foodomic analysis^54^ to circumvent issues with recording children’s diet (e.g., recall bias, calculating accurate portion size).^55^

In summary, our study is the first to report that atypical brain activity in several regions previously implicated in ASD pathophysiology mediate the relationship between tryptophan metabolites and autism severity and emotion-processing differences in ASD youth. These findings represent an important step toward mechanistic integrated models of body-brain-behavior relationships in ASD, with potential implications for future interventions. Future studies incorporating longitudinal designs focusing on critical periods of pre- and postnatal development as well as interventional designs, are needed to further explore these relationships and their relevance in ASD.

## 4. Methods

### 4.1 Participants.

Participants were recruited from healthcare clinics in Los Angeles, through advertising in the local community and social media, and by word-of-mouth. Inclusion criteria for all participants included: (a) aged 8–17 years old; (b) IQ of at least 75 on either Full-Scale Intelligence Quotient (FSIQ), or Verbal Comprehension Index (VCI) of the Wechsler Abbreviated Scale of Intelligence 2nd edition (WASI-II)^56^ c) right-handed as assessed by a questionnaire adapted from Crovitz and Zener.^57^ Exclusion criteria for all participants included: (a) history of head injury with loss of consciousness greater than 5 min; (b) not sufficiently fluent in English or parent who did not have English proficiency (as not all assessments have been validated in other languages); (c) born before 36 weeks of gestation; (d) contraindications to participating in MRI; (e) on probiotics/prebiotics for the past two weeks; and (f) on antibiotics in the past month.

Additional inclusion criteria for the NT group were: (a) no first-degree relatives diagnosed with ASD; (b) a t-score<65 on the Conners-3AI parent^58^ indicating no attention deficit hyperactivity disorder; (c) a t-score<60 on the Social Responsiveness Scale 2nd edition (SRS-2)^59^ indicating low likelihood of ASD; and (d) no psychological or neurological disorders. For the ASD group, the Autism Diagnostic Observation Schedule (ADOS-2)^60^ and the Autism Diagnostic Interview-Revised (ADI-R)^61^ were administered by a research-certified assessor to confirm ASD diagnosis. Of the ASD participants, 9 were taking antidepressants, 1 was taking anticonvulsants, 11 were on stimulants, and 3 were taking antipsychotics at the time of participation (metabolites were adjusted for medication usage, see Methods section 4.4). No NT participants were taking medication at time of participation. All participants were instructed to abstain from antibiotic usage for 30 days and probiotics for 14 days prior to participation. All participants and parents were evaluated for their capacity to give informed consent and provided their written child assent and parental consent in accordance with the study protocols approved by the University’s Institutional Review Board.

### 4.2 Behavioral Measures.

The study took place over two days. On the first day, behavioral measures and assessments were completed, and fMRI task training and desensitization took place. On the second day, participants brought their stool sample and participated in the fMRI sessions.

In addition to the screening measures, parents completed the Sensory Experiences Questionnaire (SEQ-3)^62^ to assess sensory processing and the Screen for Child Anxiety Related Emotional Disorders (SCARED-P)^63^ to measure anxiety symptoms. Participants completed the Disgust Propensity and Sensitivity Scale-Revised (DPSS-R)^64^ to assess frequency of disgusting experiences and the emotional impact of disgusting stimuli, the Alexithymia Questionnaire for Children to measure alexithymia (AQC)^65^, and the Body Perception Questionnaire-Short Form (BQP-SF)^66^ to measure interoception.

We also collected data on variables that could impact the gut microbiome. The following variables were collected from the parent or the child: birth delivery method, prenatal antibiotic usage, antibiotic and probiotic usage during infancy, gastrointestinal symptoms (GSRS), stool consistency (Bristol Stool Form Scale)^67^, sleep (Adolescent Sleep Wake Scale [ASWS]^68^; and Family Inventory of Sleep Habits [FISH]^69^, and current medication usage. For diet, parents were asked to select a diet that best reflects what their child consumes on a regular basis. We then grouped the diets into the American (high consumption of whole grains, some processed foods such as frozen and packaged foods as well as whole grain pasta and breads, limited quantities of poultry, fish, eggs and dairy, and vegetables and fruits are consumed in moderate to large quantities) or other (Mediterranean, Paleo, Vegetarian, Gluten-Free, Dairy-Free, low FODMAP, or other).

### 4.3 Stool Sample Collection

After completion of their day 1 visit, participants were given a stool collection kit (specimen cup, wooden spatula, plastic bag, Fisherbrand Scientific Commode Specimen Collection System, gloves, ice packs, and an insulated transportation container). They were instructed to collect a stool sample within 72 hours of their MRI, freeze the sample at home, and transport it in the insulated transportation container with ice packs to the lab. Once in the lab, the sample was placed in a −80° C degree freezer for storage (first at USC, and then at UCLA where they were aliquoted and stored in a −80° C degree freezer). Aliquoted samples were shipped on dry ice with a stool collection log to Metabolon for further processing and analysis on their global metabolomics and bioinformatics platform (Metabolon, 617 Davis Drive, Durham, NC).

### 4.4. Preprocessing of Metabolomic data

Peak area values were log transformed and KNN imputation was applied for missing data (Do et al., 2018). Next, data was Z score normalized and adjusted for use of antidepressants, vitamins, supplements, laxatives, antihistamines, stimulants, cognition enhancers, and antipsychotics. Specifically we regressed out significant medication/supplement effects identified using a backward selection approach (function “MASS::stepAIC” in R using the BIC, i.e., log(n) degrees of freedom). The adjusted features were then used in downstream analyses. A priori metabolite targets of interest included 26 named metabolites in the tryptophan pathway (see Figure 1).

### 4.5 Brain Imaging

#### 4.5.1 Scanning Parameters:

MRI data were acquired on a 3 Tesla MAGNETOM Prisma (Siemens, Erlangen, Germany) with a 20-channel head coil. A 5-min structural T1-weighted MPRAGE was acquired for each participant (TR = 1950 ms, TE = 3.09 ms, flip angle = 10°, 256 × 256 matrix, 176 sagittal slices, 1 mm isotropic resolution). Each functional scan consisted of an echo-planar imaging (EPI; 150 whole-brain volumes) acquired with the following parameters: TR = 2 s, TE = 30 ms, flip angle = 90°, 64 × 64 matrix, in-plane resolution 2.5 × 2.5 mm, and 41 transverse slices, each 2.5 mm thick, covering the whole-brain with a multiband factor of three. Spin Echo EPI field mapping data was also acquired in AP and PA directions with identical geometry to the EPI data for EPI off-resonance distortion correction (TR = 1,020 ms, TE1 = 10 ms, TE2 = 12.46 ms, flip angle = 90°, FOV = 224 × 224 × 191 mm3, voxel size = 2.5 mm isotropic).

#### 4.5.2 Scanning Procedure:

All participants completed a practice MRI session in a mock MRI scanner prior to the fMRI tasks to become familiarized with the task and the MRI environment and to increase comfortability and minimize head motion. Functional MRI procedure, task stimuli, fMRI acquisition, and data preprocessing were completed following the protocol previously published in Kilroy et al., 2021.^38^ We utilized a head-motion cut-off of absolute FD>1.5 mm. Five participants (4 ASD, 1 NT) were excluded for head motion in the watching facial expressions and hand actions task, 3 (2 ASD, 1 NT) for disgust processing, and 2 (1 ASD, 1 NT) from watching others being touched. There were no significant differences in relative head motion between the two groups for the disgust processing (*t*=0.981, *p*=0.33) and watching others being touched (*t*=−1.029, *p*=0.307) tasks, but significant differences were present for the observation of facial expressions and hand actions task (*t*=−2.572, *p*=0.015). Please see *fMRI processing* in Section 4.6.2 for details regarding motion correction.

#### 4.5.3 fMRI Tasks:

The task-based fMRI paradigms were selected based on existing literature showing significant ASD vs. NT differences during these tasks, their relevance to key ASD symptomatology (socio-emotional processing and sensory sensitivities^38,49,70,71^, and/or their relevance to vagally mediated emotional processes (disgust and emotion processing).^42^ The fMRI tasks included: watching videos of facial expressions/body actions, physical and social disgust processing tasks, and watching videos of others being touched (*Supplemental Figure 1*). Stimuli were presented using the Psychophysics Toolbox^72^ on MATLAB. During all tasks, participants were instructed to simply watch all videos and remain as still as possible. fMRI tasks are described in A-C below.

##### A. Watching videos of facial expressions and hand actions (n=78; 38 NT [19 female, 19 male], 40 ASD [11 female, 29 male]):

One 9-minute run with five 15-sec blocks of video-stimuli were shown. As *Supplemental Figure 1A* shows, blocks consisted of one of three categories of stimuli: emotional facial expressions (e.g., happy expression), non-emotional facial expressions (e.g., tongue to lip), or bimanual hand actions (e.g., playing the xylophone). Each video was presented for 3.75 sec followed by a 1.25 sec black screen between each stimulus, there were 3 videos per block, and both male and female actors were included in each block. For further details on stimuli, please see Kilroy et al., 2021.^38^

##### B. Disgust Processing (n=46; 22 NT [12 female, 10 male], 24 ASD [6 female, 18 male]):

There were four categories of stimuli, disgusting foods, disgusted facial expressions, neutral foods, and neutral facial expressions (*Supplemental Figure 1B*). The neutral and the disgusted facial expressions were chosen from an online repository (NimStim)^73^ and from previous research (EmStim)^38^ then edited and counterbalanced so that each participant saw the same actor displaying a neutral and disgusted facial expression. To ensure that the neutral food images were indeed items the participant truly had no preferential or disgusting feelings for, all participants were administered a questionnaire prior to participating in the study, to assess their preferences for each food stimuli. For each participant, 18 images were used from each stimulus category. One fMRI run was presented to all participants, including six blocks per stimulus category. Within each 15-sec block, three different images from the same category were presented with a 250-ms fixation crosshair between each stimulus (e.g., three different disgusting food images). Thus, the fMRI task consisted of 24 blocks (5 per stimulus category), lasting for a single 10-min run. For further details on stimuli, please see Jayashankar et al., *in review*.^42^

##### C. Watching others being touched (n=37; 19 TD [11 female, 8 male], 18 ASD [5 female, 13 male]):

Participants watched four different videos where a person strokes the arm of another person in the MRI scanner with: 1) their hand with glove on (social touch), 2) a dry sponge (object touch), 3) their hand with glove on hovering next to the person’s arm (social touch control), and 4) a dry sponge hovering next to the person’s arm (object touch control) following asimilar to the protocol used in Green et al., 2015 (*Supplemental Figure 1C*).^74^ Each video was 15 sec long followed by a 15 sec rest block. During the rest blocks, participants were shown a black crosshair in the middle of a white screen. Excluding an initial junk block, five blocks of each stimulus condition were alternated with rest in a pseudo-random sequence. Stimuli conditions were counterbalanced across participants. We note that our original intention was to physically touch participants while in the scanner, but as this run was largely conducted during the initial period of the COVID-19 pandemic, we were obliged to remain at a 6-ft distance from our participants, and thus used videos of touch instead, as this has previously been shown to show strong effects.^75,76^

### 4.6 Analysis

#### 4.6.1 Behavioral Group Differences.

Independent samples t-tests and Fisher’s exact tests were conducted to determine ASD-NT differences in demographic and behavioral variables. Significance was set to p<.05.

#### 4.6.2 fMRI

##### fMRI Preprocessing:

All analyses followed best practices in fMRI analysis, as detailed in our prior studies.^38^ The data analytic approach used to address each of our research questions utilized FMRIB’s Software Library 6.0 (FSL).^77–81^ Standard preprocessing pipeline was performed involving: (a) structural T1 brain extraction and non-brain tissue removal; (b) smoothing with 5 mm FWHM Gaussian kernel; (c) B0 unwarping along y-axis; (d) high pass filter with 100 sec cutoff; (e) realignment using MCFLIRT to obtain motion estimates; and (f) Independent component analysis (ICA). Preprocessed data was fed into the ICA AROMA algorithm^82^, which filtered out noise and motion components from the whole brain signal. Registration to the MNI-152 standard atlas using 12 degrees-of-freedom affine transformation and FNIRT nonlinear registration were performed.^78,79^

###### Within-group analyses:

Individual participants’ statistical images were subjected to higher-level mixed-effects analyses using FSL’s FLAME Stage 1 algorithm, modeling the stimulus conditions for each participant as separate regressors. For watching facial expressions and hand actions, regressors included: emotional faces, non-emotional faces, and bimanual hand actions. For disgust processing, regressors included: disgusting foods, neutral foods, disgusted facial expressions, neutral facial expressions. Subject-specific head motion parameters were used as nuisance regressors. For observation of others being touched, regressors included social touch and object touch.

###### Between-group Analysis:

Between-group comparisons between the NT and ASD groups were performed using higher level mixed-effects analyses with FSL’s FLAME 1 algorithm. Age, Sex, and IQ were used as covariates in all group-level analyses. For watching facial expressions and hand actions, groups were contrasted on: all stimuli>rest; emotional facial expressions>rest; non-emotional facial expressions>rest; all facial expressions>rest; hand actions>rest. For disgust processing, groups were contrasted on: disgusting foods>rest, disgusted faces>rest. For observation of others being touched, groups were contrasted on: social touch>rest and object touch>rest. For the facial expressions/hand action task and the disgust tasks, the resulting group-level images for all models were thresholded at voxel Z>3.1, with a cluster size probability correction threshold of *p*<.05. For observation of others being touched, a more lenient threshold (Z > 2.3 cluster size probability threshold of *p*<.05) was used to have more sensitivity to detect effects given the more subtle observation (rather than physical touch) task used, due to COVID-19 restrictions (see Methods 4.5.3.C). In addition, for disgust and facial expression/hand action tasks, for hypothesized regions of interest (ROIs), a small volume correction (SVC) analysis with a significance threshold of *p*<0.01 using predefined masks for disgust and observation tasks. For the facial expression/hand action task, we used structurally defined anterior insula parcellations from extant literature and the Harvard-Oxford atlas parcellations for the pACC and amygdala. For the IFGop, we used a hand-drawn anatomically derived ROI^38^ and previously published insula parcellations.^83^ For the disgust task, ROIs for SVC analysis were defined utilizing the Neurosynth database (which performs automated large-scale meta-analyses of fMRI data), using the search terms - “disgust”, “emotional faces”, and “food”, and we also included insula parcellations from extant literature.^83^ Functional ROIs were then masked with structural ROIs from the Harvard-Oxford atlas (thresholded at 30% probability) to ensure they captured non-overlapping regions.

### 4.4 General Linear Models (GLMs).

GLMs were applied within the ASD group and across groups to test brain-behavior, brain-metabolite, and metabolite-behavior relationships.The GLMs included group as a factor, and sex, age, IQ and BMI were included as covariates. As a measure of effect size, we report the standardized beta (Std β). Std β between 0.10–0.29 is considered small, 0.30–0.49 medium, and greater than 0.50 large.^84^ Brain ROIs were chosen based on group differences in fMRI tasks as well as prior studies supporting atypicalities in brain activity in the chosen ROIs.^38,42,49,70,71^ The Benjamini-Hochberg method to correct for multiple comparisons was used; the false discovery reporting threshold set at 10% (FDR).^85^ We used FDR correction for the number of dependent variables in each analyses. Specifically for brain-metabolite and brain-behavior analyses, FDR correction was for the number of ROIs compared. For metabolite-behavior analyses, FDR correction was for the number of metabolites compared. To limit the number of comparisons, only metabolites that significantly correlated with brain activity were included in the metabolite-behavior analyses.

### 4.5 Mediation Models

Exploratory mediation analyses were conducted to determine if the brain was a mediator of the relationship between metabolite and behavior in the ASD group. The variables included in the mediation models were selected based on metabolites and behaviors that had significant associations with the same task-based ROIs. Mediation modeling was performed using lavaan in R. We estimated the bootstrapped 95% percentile confidence intervals for the indirect effects.^86^ Confidence intervals that do not contain zero are considered significant. Because age, sex, and BMI are collinear, we ran analyses using only age and FSIQ as covariates. Additionally, in terms of regressors for brain and behavior (as opposed to metabolites), it is less relevant to control for BMI.
